# Hsp104-dependent ability to assimilate mannitol and sorbitol conferred by a truncated Cyc8 with a C-terminal polyglutamine in *Saccharomyces cerevisiae*

**DOI:** 10.1371/journal.pone.0242054

**Published:** 2020-11-11

**Authors:** Hideki Tanaka, Kousaku Murata, Wataru Hashimoto, Shigeyuki Kawai

**Affiliations:** 1 Laboratory of Basic and Applied Molecular Biotechnology, Division of Food Science and Biotechnology, Graduate School of Agriculture, Kyoto University, Uji, Kyoto, Japan; 2 Faculty of Science and Engineering, Department of Life Science, Setsunan University, Ikeda-Nakamachi, Neyagawa, Osaka, Japan; 3 Laboratory for Environmental Biotechnology, Research Institute for Bioresources and Biotechnology, Ishikawa Prefectural University, Suematsu, Nonoichi, Ishikawa, Japan; University of Parma, ITALY

## Abstract

Tup1-Cyc8 (also known as Tup1-Ssn6) is a general transcriptional corepressor. D-Mannitol (mannitol) and D-sorbitol (sorbitol) are the major polyols in nature. Budding yeast *Saccharomyces cerevisiae* is unable to assimilate mannitol or sorbitol, but acquires the ability to assimilate mannitol due to a spontaneous mutation in *TUP1* or *CYC8*. In this study, we found that spontaneous mutation of *TUP1* or *CYC8* also permitted assimilation of sorbitol. Some spontaneous nonsense mutations of *CYC8* produced a truncated Cyc8 with a C-terminal polyglutamine. The effects were guanidine hydrochloride-sensitive and were dependent on Hsp104, but were complemented by introduction of *CYC8*, ruling out involvement of a prion. Assimilation of mannitol and sorbitol conferred by other mutations of *TUP1* or *CYC8* was guanidine hydrochloride-tolerant. It is physiologically reasonable that *S*. *cerevisiae* carries this mechanism to acquire the ability to assimilate major polyols in nature.

## Introduction

Polyols are acyclic sugar alcohols that are widely distributed in bacteria, fungi, algae, and higher plants [1]. D-Mannitol (mannitol) is the most abundant polyol in nature [2]. In brown macroalgae, mannitol is one of the main carbohydrates and may be a carbon source for production of fuels and chemicals [3]. D-Sorbitol (sorbitol) is the major photosynthate in *Rosaceae* species, including apple, pear, and loquat [4, 5]. Despite the wide distribution of mannitol in nature, the budding yeast *Saccharomyces cerevisiae* is generally thought to be unable to utilize mannitol as a carbon source, although *S*. *cerevisiae* has two genes (*DSF1* [or *MAN1*] and *YNR073C* [or *MAN2*]) that code for mannitol 2-dehydrogenase, which oxidizes mannitol to fructose, and 3 genes (*HXT13*, *HXT15*, and *HXT17*) encoding transporters for mannitol and sorbitol [6–12]. Moreover, *S*. *cerevisiae* seems not to utilize sorbitol, since most strains of *S*. *cerevisiae* exhibit an unusually long lag period of 2–4 weeks for growth in a medium containing sorbitol as the sole carbon source [13]. *S*. *cerevisiae* also has two genes for sorbitol 2-dehydrogenases (*SOR1* and *SOR2*) that are needed for sorbitol metabolism and have 99% identity at the nucleotide level [12].

**Fig 1:**
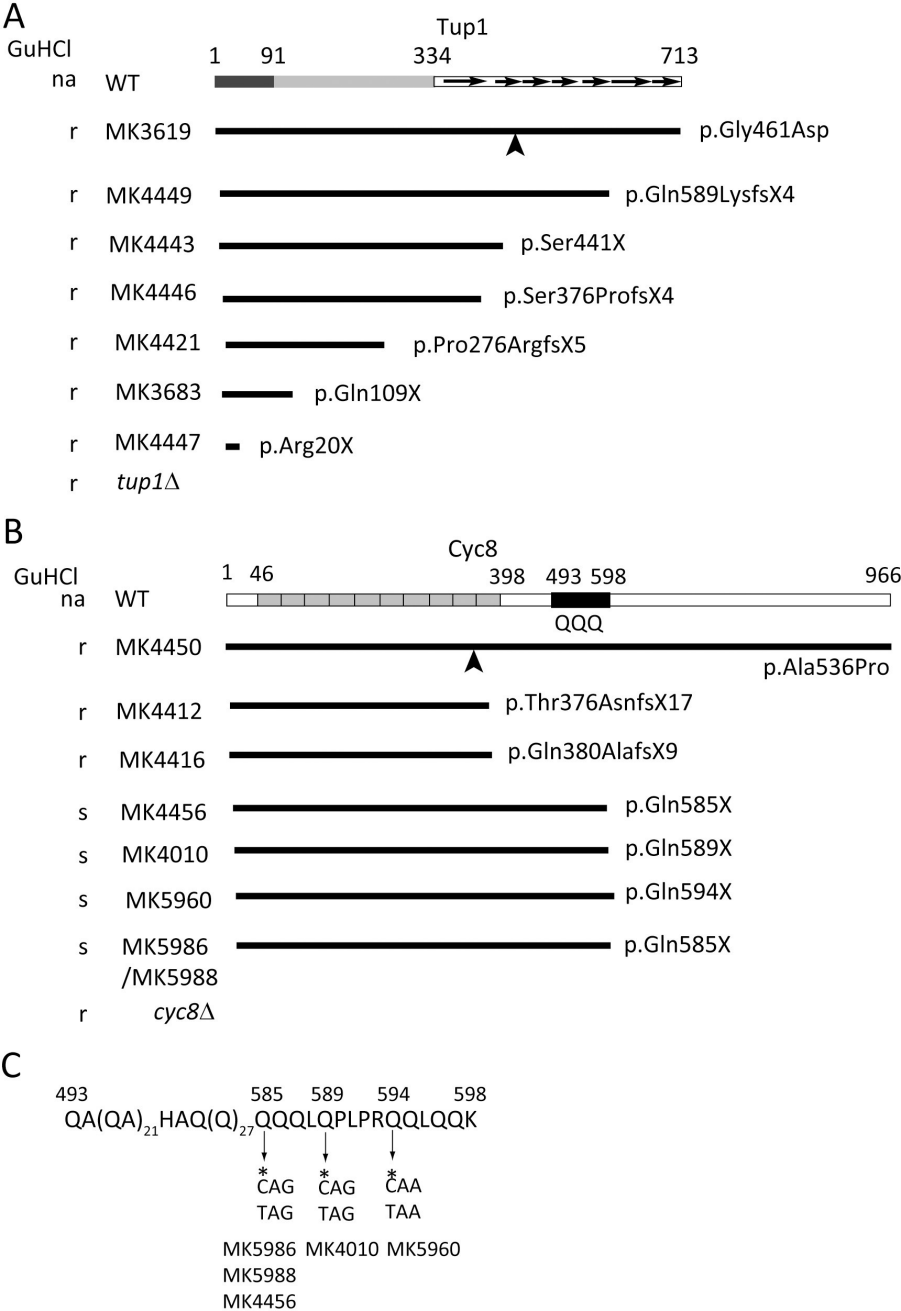
Structures of Tup1 and Cyc8. A: Schematic structure of Tup1 (713 amino acids) [10]. N-terminal (dark gray, residues 1–91), central (light gray), and C-terminal (white, 334–713) domains are shown. WD-repeat motifs are represented as arrows [14]. B: Schematic structure of Cyc8 (966 amino acids) [10]. TPR motifs are shaded [15]. The Q-rich region (493–598) is shown in black and marked with QQQ. The mutated sites in MK3619 and MK4450 are indicated by arrowheads and the truncated structures of Tup1 and Cyc8 in each strain are indicated by bold lines (A, B). Guanidine hydrochloride (GuHCl) resistance and sensitivity are shown by r and s, respectively. na: not applicable. WT: BY4742. C: Details of the C-terminal Q-rich region (493–598) in Cyc8. The C residues of the codons (CAG and CAA) for Gln-585, Gln-589, and Gln-594 are marked by asterisks and substituted to T residues by nonsense mutations resulting in stop codons (TAG and TAA).

We have shown that *S*. *cerevisiae* BY4742 wild type (WT) acquires the ability to assimilate mannitol during prolonged cultivation on solid or liquid synthetic mannitol (SM) medium containing mannitol as the sole carbon source due to a spontaneous mutation in the genes for the Tup1-Cyc8 corepressor (Fig 1A and 1B) [10]. We refer to strains with the ability to utilize mannitol as Mtl+ strains [10]. These include MK3619 and MK3683 strains, both of which have a mutation in *TUP1* (*tup1* c.1382G>A and *tup1* c.325C>T, resulting in p.Gly416Asp and p.Gln109X), and MK4412 and MK4416 strains, which have a *CYC8* mutation (*cyc8* c.1129_1138del and *cyc8* c.1139_1164del, resulting in p.Thr376AsnfsX17 and p.Gln380AlafsX9) [10] (Fig 1A and 1B). Microarray analysis revealed induced transcription of the genes for mannitol 2-dehydrogenase and mannitol transporters (*DSF1*, *HXT15*, and *HXT17*) in the Mtl+ (MK3619 and MK3683) strains grown in SM liquid medium, in accord with the two strains being able to assimilate mannitol [10]. Microarray analysis also showed transcription of *SOR1/2* [12], suggesting that these Mtl+ strains can also assimilate sorbitol due to the *TUP1* or *CYC8* mutation.

Tup1-Cyc8 (also known as Tup1-Ssn6) is a general transcriptional corepressor [16, 17] that is composed of four molecules of Tup1 and one Cyc8 [18]. Tup1-Cyc8 is implicated in repression of over 300 genes, including cell-type specific, glucose-repressible, hypoxic, DNA damage-inducible, and flocculation genes [19, 20]. Tup1 consists of 713 amino acids (Fig 1A) and Cyc8 has 966 amino acids and contains a central glutamine (Q)-rich region comprising poly(QA) [(QA)_22_ (residues 493–536) and (QA)_9_ (539–556)] and polyQ [(Q)_31_ (557–587) and (589–598)] (Fig 1B and 1C). Patel *et al*. showed that transient overexpression of the C-terminal region of Cyc8 (465–966) including the Q-rich region in a *S*. *cerevisiae cyc1Δ/cyc1Δ* diploid strain induced a prion form of Cyc8, [*OCT*^+^] [21]. Moreover, a computationally predicted putative prion domain of Cyc8 (443–672) again encompassing the Q-rich region behaved biochemically like a prion when this domain was overexpressed, with formation of a fluorescent foci and intracellular aggregates [22]. Prions are usually eliminated by guanidine hydrochloride (GuHCl) treatment [21, 23], and the Hsp104 chaperone is needed for heritability of all known yeast prions. A millimolar concentration of GuHCl in media largely removes prion elements and the prion-induced phenotype from yeast cells due to inhibition of the activity of Hsp104 [21, 24, 25].

In this study, we found that a spontaneous mutation in *TUP1* or *CYC8* conferred the ability to assimilate both mannitol and sorbitol due to dysfunction of Tup1-Cyc8. In many cases, the Mtl+ and Sor+ phenotype was tolerant to GuHCl treatment. Moreover, we spontaneously obtained *S*. *cerevisiae* strains with the Mtl+ and Sor+ phenotype that were sensitive to GuHCl and strictly dependent on Hsp104. Acquisition of the Hsp104-dependent Mtl+ and Sor+ phenotype was attributable to dysfunction of the truncated Cyc8 with polyQ at its C-terminus.

## Materials and methods

### Plasmids

Primers used in the study are listed in Table 1. Genomic PCR was conducted using KOD-Plus Neo (Toyobo, Japan), unless otherwise stated. *HSP104* was amplified from genomic DNA of *S*. *cerevisiae* BY4742 with PCR using primers 1 and 2 and inserted into SalI sites of pESC-URA plasmid (pESC-U; Agilent), resulting in pESC::*HSP104* (pMK5659) to allow galactose-induced overexpression of the target gene. Lys-218 and Lys-620 of Hsp104 encoded in pMK5659 were substituted to Thr with inverse PCR using pMK5659 as a template and primers 3–6, yielding pESC::*HSP104_KT* (pMK5680) to express a dominant-negative *HSP104-KT* allele encoding mutated Hsp104 with both ATP-binding sites inactivated [25]. *HSP104* was amplified from genomic DNA of BY4742 with PCR using primers 7 and 8 and inserted into SmaI sites of pRS415 [26], resulting in pRS415::*HSP104* (pMK6284). Sequences of the cloned genes were confirmed.

**Table 1:** Primers used in the study.

No.	Name	Sequence	Description
**1**	pESC-U_S16HSP104F	*GGGCCCGGGC**GTCGAC*ATGAACGACCAAACGCAATTTAC	Amplification of *HSP104*
**2**	pESC-U_S16HSP104R	*TCTGTTCCATGTCGAC*TTAATCTAGGTCATCATCAATTTC	Amplification of *HSP104*
3	HSP104_A653C_F	TGAGCCAGGTATCGGTACGACCGCTATTATTGAAG	Site-directed mutation
4	HSP104_A653C_R	CTTCAATAATAGCGGTCGTACCGATACCTGGCTCA	Site-directed mutation
5	HSP104_A1859C_F	GTTTGTCCGGTTCCGGTACAACTGAATTGGCTAAAAAAG	Site-directed mutation
6	HSP104_A1859C_R	CTTTTTTAGCCAATTCAGTTGTACCGGAACCGGACAAAC	Site-directed mutation
7	SI_15_HSP104_-714_-695F	*GAATTCCTGCAGCCC*TAGAGTTAGCGCTAGAAACC	Amplification of *HSP104*
8	SI_15_HSP104_+943_+924R	*ACTAGTGGATCCCCC*TATGAGAAGCTGTCATCGAG	Amplification of *HSP104*
9	HSP104_-714_-695F	TAGAGTTAGCGCTAGAAACC	Amplification of *HSP104*
10	HSP104_+943_+924R	TATGAGAAGCTGTCATCGAG	Amplification of *HSP104*

Sequences needed for in-fusion are shown in italics. Sites for restriction enzymes and substitutions are underlined.

### Strains

*S*. *cerevisiae* strains used in the study are listed in Table 2. *Escherichia coli* strain DH5α was used for plasmid construction. Transformation of yeast cells with plasmid DNA was conducted as described elsewhere [27]. MK6331 and MK6140 strains were constructed by replacing *HSP104* in MK4010 and MK4416 strains with *kanMX4*, which was amplified from genomic DNA of MK6093 strain with PCR using primers 9 and 10.

**Table 2:** *S*. *cerevisiae* strains used in the study.

Strain	Description	Source
**BY4742**	MATα *his3Δ1 leu2Δ0 lys2Δ0 ura3Δ0*	Euroscarf
**MK4010**	BY4742 *cyc8* c.1765C>T	[10]
**MK4412**	BY4742 *cyc8* c.1129_1138del	[10]
**MK4416**	BY4742 *cyc8* c.1139_1164del	[10]
**MK4450**	BY4742 *cyc8* c.1066C>T	[10]
**MK4456**	BY4742 *cyc8* c.1752G>A, 1753C>T	[10]
**MK4965**	BY4742 *cyc8Δ*::*kanMX4*	Euroscarf
**MK3619**	BY4742 *tup1* c.1382G>A	[10]
**MK4449**	BY4742 *tup1* c.1765delC	[10]
**MK4443**	BY4742 *tup1* c.1322C>A	[10]
**MK4446**	BY4742 *tup1* c.1122_1132del	[10]
**MK4421**	BY4742 *tup1* c.824_839del	[10]
**MK3683**	BY4742 *tup1* c.325C>T	[10]
**MK4447**	BY4742 *tup1* c.58A>T	[10]
**MK4035**	BY4742 *tup1Δ*::*kanMX4*	Euroscarf
**MK5753**	MK4010 pESC-U	This study
**MK5755**	MK4010 pESC-U::*HSP104_KT* (pMK5680)	This study
**MK6089**	MK4412 pESC-U	This study
**MK6088**	MK4412 pESC-U::*HSP104_KT* (pMK5680)	This study
**MK6011**	MK4416 pESC-U	This study
**MK6012**	MK4416 pESC-U::*HSP104_KT* (pMK5680)	This study
**MK6091**	MK4456 pESC-U	This study
**MK6090**	MK4456 pESC-U::*HSP104_KT* (pMK5680)	This study
**MK5992**	MK5960 pESC-U	This study
**MK5993**	MK5960 pESC-U::*HSP104_KT* (pMK5680)	This study
**MK5996**	MK5988 pESC-U	This study
**MK5997**	MK5988 pESC-U::*HSP104_KT* (pMK5680)	This study
**MK6093**	BY4742 *hsp104Δ*::*kanMX4*	Euroscarf
**MK6331**	MK4010 *hsp104Δ*::*kanMX4*	This study
**MK6140**	MK4416 *hsp104Δ*::*kanMX4*	This study
**MK6345**	MK6331 pRS415	This study
**MK6344**	MK6331 pRS415::*HSP104* (pMK6284)	This study

### Media and cultivations

Standard yeast media were used [28]. YPD and YPG media consisted of YP [2% (w/v) yeast extract and 2% (w/v) tryptone, pH 5.6] with 2% glucose and 3% glycerol, respectively. Adenine was supplemented to 14.7 mg/L in YPD, resulting in YPDA. Media were solidified with 2% (w/v) agar. GuHCl stock solution (2.0 M in pure water) was sterilized with a filter and added to the autoclaved YPDA medium to 5 mM. Other media consisted of 0.67% (w/v) yeast nitrogen base without amino acids (YNB, pH 5.6) (BD) and dropout supplement -Ura (Clontech) plus 20 mg/L Ura with 2% (w/v) glucose (SC), 2% (w/v) mannitol (SM), 2% (w/v) sorbitol (SS), or 2% (w/v) galactose plus 1% (w/v) raffinose (SGR). For selective SC-U, SM-U, or SGR-U medium, Ura was not included. Yeast strains were aerobically grown at 30°C. For measurement of growth, cells grown on YPG solid medium were suspended in sterilized pure water (SPW; Elix, Millipore) and inoculated into 1 ml SM, SS, or SC medium in a test tube at an OD_600_ of 0.05, unless otherwise stated. Strains were grown at 145 strokes per min (spm) in a Personal Lt-10F (Taitec, Japan). In the case of flocculated cells, OD_600_ was measured after the culture was mixed with 0.1 volume of 500 mM EDTA. The ρ^+^ strains having intact mitochondrial genomes [28, 29] were confirmed through their growth on YPG solid medium. Anaerobic growth was conducted using an AnaeroPack (Mitsubishi Gas Chemical). Strains were stored at -80°C in the presence of 17% (v/v) glycerol.

### Expression of a dominant-negative *HSP104-KT* allele

Strains were grown in YPDA liquid medium and transformed with each of pESC-U and pMK5680 (pESC-U::*HSP104_KT*) using selective SM-U solid medium. Each transformant was grown in SM liquid medium and stored at -80°C in the presence of 17% glycerol. The stock strain was pre-cultured on SM-U solid medium, inoculated in 1.0 mL SGR-U liquid medium to OD_600_ of 0.05, and grown for 2 days. Cells in cultures were washed twice with SPW and then suspended in SPW, inoculated in SC-U, SM-U, or SS-U liquid medium to OD_600_ of 0.05, and further cultivated for 2 days.

### Obtaining naturally occurring strains that assimilate mannitol and sorbitol

*S*. *cerevisiae* BY4742 WT strain was grown in YPDA liquid medium for 24 h, collected, and washed with SPW twice. The washed cells (approximately 10^7^) were spread on SM solid medium and grown for more than 7 days. Colonies were purified once on SM solid medium, resulting in Mtl+ strains [10]. Similarly washed cells (approximately 10^7^) were also spread, grown, and purified once on SS solid medium, yielding Sor+ strains. BY4742 WT cells carrying YEplac195 were also grown in SC-U liquid medium and treated as above, but using SM-U or SS-U solid medium.

## Results

### *S*. *cerevisiae* acquires the ability to assimilate sorbitol due to mutation of *TUP1* or *CYC8*

Several *S*. *cerevisiae* strains with the ability to assimilate mannitol in a BY4742 background were obtained due to a spontaneous mutation in *TUP1* or *CYC8* [10]. Complete deletion of *TUP1* or *CYC8* also conferred the ability to assimilate mannitol [10]. The strains with this ability are referred to as Mtl+ strains (Fig 1: MK3619, MK4449, MK4443, MK4446, MK4421, MK3683, MK4447, *tup1Δ*, MK4450, MK4412, MK4416, MK4456, MK4010, and *cyc8Δ* strains), while strains without this capacity are Mtl- strains [10]. The MK4010 strain has been reported to carry no mutation in *TUP1* or *CYC8* [10]. However, in this study, we resequenced *CYC8* in the MK4010 strain and identified a c.1765C>T mutation. This introduces a nonsense amber mutation (CAG to TAG) and results in a truncated Cyc8 with a C-terminal polyQ (Fig 1C). It should be noted that the MK4456 strain carries c.1752G>A (a synonymous substitution) and c.1753C>T (a nonsense amber mutation resulting in CAG to TAG) in *CYC8* and also results in a truncated Cyc8 with polyQ at its C-terminus (Fig 1C).

**Fig 2:**
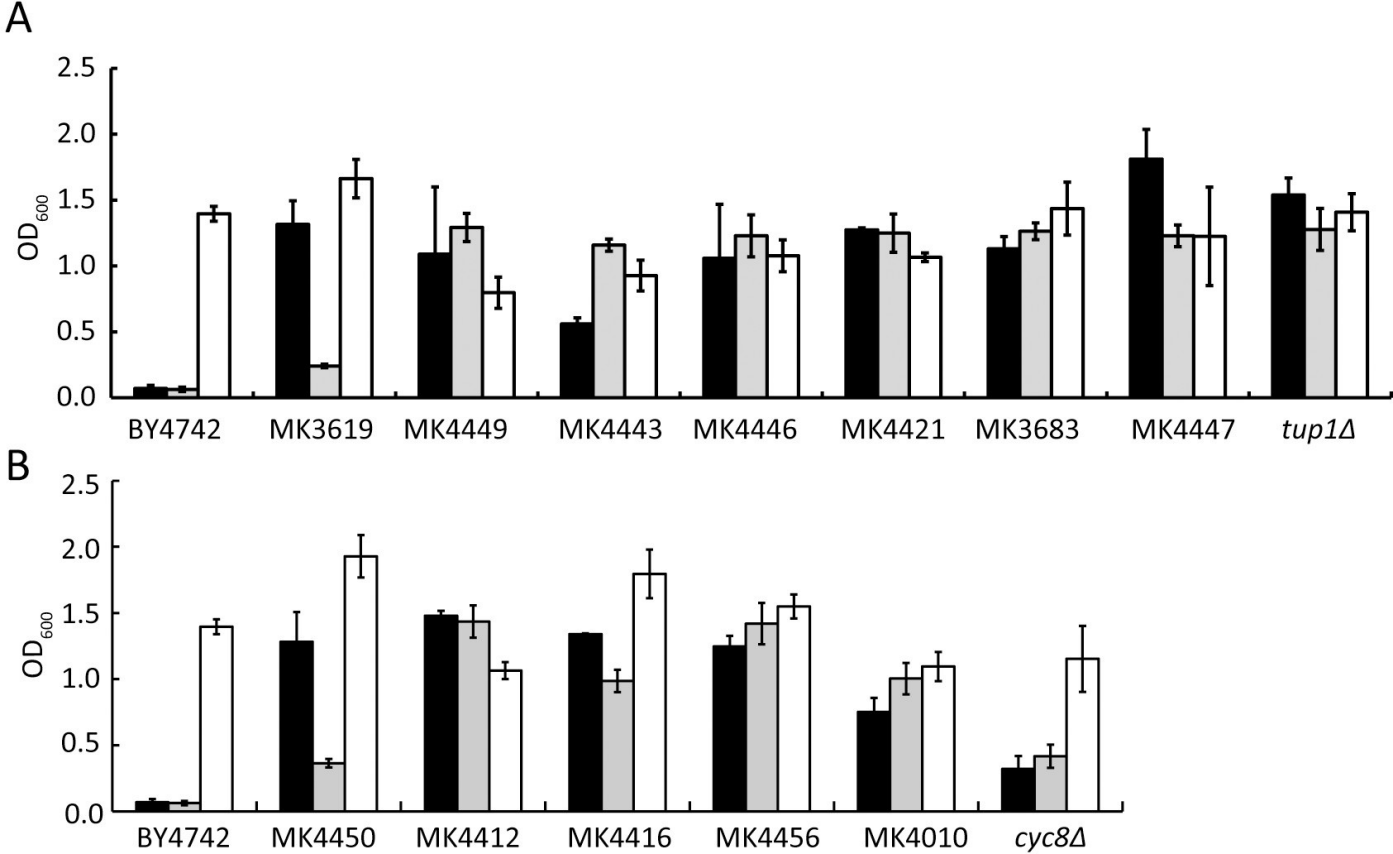
Growth of Mtl+ strains in SM, SS, and SC media. The parental BY4742 strain and Mtl+ strains due to *TUP1* mutation (A) and *CYC8* mutation (B) were pre-cultured on YPG solid medium, inoculated into 1.0 mL SM (black bar), SS (gray bar), and SC (white bar) to OD_600_ of 0.025, and cultivated aerobically for 2 days. Only the *cyc8Δ* strain was pre-cultured on YPD solid medium due to its inability to grow on YPG solid medium [10]. Data are shown as averages and standard deviations (n = 3).

Microarray analysis of Mtl+ strains showed induced transcription of *SOR1/2* encoding sorbitol dehydrogenase and of *HXT15* and *HXT17* encoding transporters for mannitol and sorbitol [10, 12], which suggests that the Mtl+ strain had also acquired the capacity to assimilate sorbitol. As expected, the Mtl+ strains, including the *tup1Δ* and *cyc8Δ* strains, were able to grow in liquid synthetic SS medium containing sorbitol as the sole carbon source, while the parental WT strain could not do so (Fig 2). Strains with and without the ability to assimilate sorbitol are referred to as Sor+ and Sor- strains, respectively.

The phenotype that did not grow in SM and SS media (Sor- and Mtl- phenotype) was specific to liquid medium because a BY4742 ρ^+^ strain with this phenotype in liquid medium was able to form a colony of size similar to MK4416 strain, but smaller than MK4412 strain, on solid SS and SM media, in which MK4412 and MK4416 strains show the phenotype that grows in liquid SM and SS media (Fig 2 and S1 Fig). As for Mtl+ strains [10], Sor+ strains required functional mitochondria and respiration to assimilate sorbitol (S2A Fig); the ρ^+^ phenotype was again a prerequisite for assimilation of sorbitol.

To check if Sor+ strains could be obtained spontaneously, as for Mtl+ strains, BY4742 WT cells (10^7^ cells) were spread on a solid SM or solid SS medium. After cultivation for 7 days, several colonies were visible on each medium (S3A Fig). The 7 colonies (Nos. 1 to 7) on solid SS medium were purified once on the same medium, and the 5 resultant strains were able to grow in liquid SM and SS media (Mtl+ and Sor+ phenotype) (Nos. 1, 2, 3, 4, 7; S3B Fig). Similarly, the 7 colonies (Nos. 11 to 17) on solid SM medium were purified and the 3 resultant strains also had the Mtl+ and Sor+ phenotype (Nos. 11, 14, 15; S3B Fig). The Mtl+ strains obtained from parental Mtl- strains (BY4741, AH109, NV191, SEY6210) [10] were also able to assimilate sorbitol. These results show that *S*. *cerevisiae* spontaneously acquires the ability to assimilate sorbitol, as well as mannitol, due to a spontaneous mutation in *TUP1* or *CYC8*. It should be noted that the *tup1Δ* and *cyc8Δ* strains also exhibited the ability to assimilate mannitol [10] and sorbitol (Fig 2).

### Truncated Cyc8 with C-terminal polyQ confers a Hsp104-dependent Mtl+ and Sor+ phenotype in *S*. *cerevisiae*

**Fig 3:**
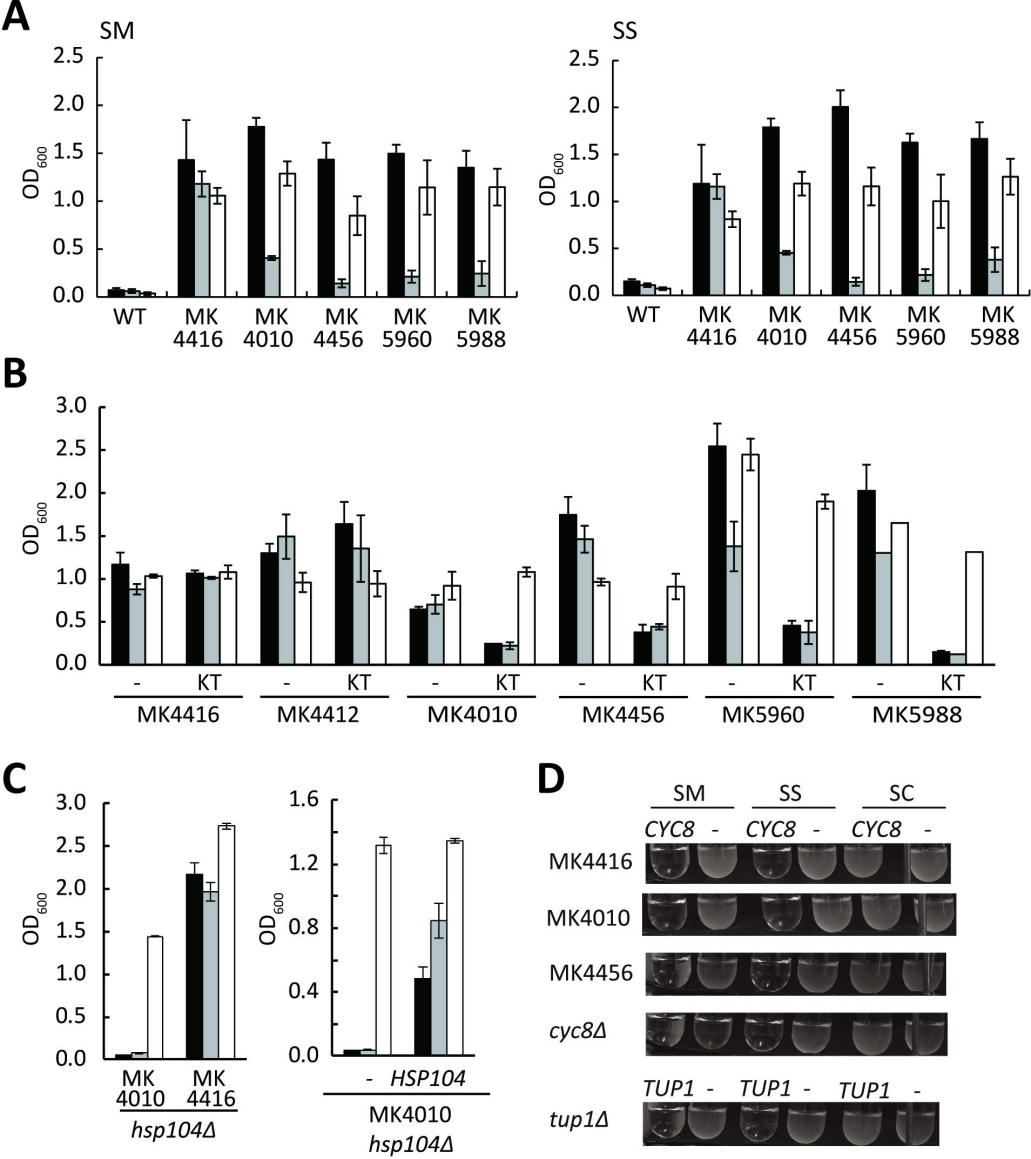
Reversible GuHCl-sensitive growth phenotype of MK4010 strain. A: A single colony of the indicated strain grown on YPD solid medium was cultivated in 1.0 mL SM (left) or SS (right) medium (as in A) for 2 days (black bar). The rest of the same colony was treated with GuHCl and the resultant GuHCl-treated strains were cultivated in SM or SS medium for 2 days (gray bar). The GuHCl-treated strains were again restreaked onto YPD solid medium and the resultant strains were cultivated in SM or SS medium for 2 days (white bar). B: Overexpression of a dominant-negative *HSP104-KT* allele eliminated the Mtl+ and Sor+ phenotype. Transformants of the indicated hosts with an empty plasmid (pESC-U, -) or pESC-U::*HSP104_KT* (KT) were grown in 1.0 mL of SM-U (black bar), SS-U (gray bar), or SC-U (white bar) medium for 2 days, as described in Materials and Methods. C: Deletion of *HSP104* eliminated the Mtl+ and Sor+ phenotype. MK4010 *hsp104Δ* ρ^+^ (MK6331) and MK4416 *hsp104Δ* ρ^+^ (MK6140) strains were grown in 1.0 mL of SM-U (black bar), SS-U (gray bar), or SC-U (white bar) medium for 2 days (left). Transformants of MK4010 *hsp104Δ* ρ^+^ (MK6331) strain with pRS415 (-) or pRS415::*HSP104* (pMK6284; *HSP104*) were grown in 1.0 mL of SM-L (black bar), SS-L (gray bar), or SC-L (white bar) medium for 2 days. Data are shown as averages and standard deviations (n = 3). (A, B, C). D: Complementation of growth in SM, SS, and SC media of the indicated strains by introduction of *CYC8* (YCplac33-*CYC8*, pKS292) or *TUP1* (YCplac33-*TUP1*, pKS291) [10] and empty vector (-: YCplac33). Cells were pre-cultured on SC-U solid medium, inoculated into 1.0 mL SM, SS, and SC media to OD_600_ of 0.05, and cultivated aerobically for 2 days. Growth of the *cyc8Δ* strain with an empty vector was low, as described previously [10].

We examined if the Mtl+ and Sor+ strains (Fig 1) lost the Mtl+ and Sor+ phenotype after GuHCl treatment (i.e., after growth on solid YPD medium containing 5.0 mM GuHCl, which inhibits the Hsp104 activity needed for heritability of yeast prions [21, 24, 25]), due to previous findings of a relationship of Cyc8 with prions [21, 22]. Among the tested strains, only MK4010 and MK4456 carrying a mutation in *CYC8* showed GuHCl sensitivity (Fig 1B, S4 Fig); i.e. GuHCl-treated MK4010 and MK4456 strains had the Mtl- and Sor- phenotype. Again, MK4010 and MK4456 strains carry a mutated *CYC8* encoding the truncated Cyc8 with C-terminal polyQ (Fig 1B). The GuHCl-treated MK4010 and MK4456 strains with the Mtl- and Sor- phenotype were verified as a ρ^+^ strain. The Mtl+ strain (MK4416) carrying a mutation in *CYC8* (cyc8 c.1139_1164 del, Fig 1B) had the Mtl+ and Sor+ phenotype even after GuHCl treatment, and thus MK4416 was GuHCl-tolerant (Fig 3A, S4A Fig). The Mtl- and Sor- phenotype of the GuHCl-treated MK4010 and MK4456 strains reverted to the Mtl+ and Sor+ phenotype after streaking on YPD solid medium (Fig 3A). Thus, the Mtl+ and Sor+ phenotype of the MK4010 and MK4456 strains, but not of the MK4416 strain, was reversed by GuHCl.

Among the 8 strains with the Mtl+ and Sor+ phenotype obtained in the study (S3 Fig), only one (S3 Fig, colony No. 2) showed GuHCl sensitivity (Fig 3A, S4B Fig). This strain was named MK5960. Among the newly obtained 13 Mtl+, 2 were GuHCl-sensitive and were named MK5986 and MK5988 (S4C Fig). All three GuHCl-sensitive strains (MK5960, MK5986, and MK5988) carried nonsense mutations in *CYC8*: ochre (CAA to TAA; c.1780C>T) in the MK5960 strain and amber (CAG to TAG; c.1753C>T) in the MK5986 and MK5988 strains. These mutations all result in a truncated Cyc8 with polyQ at its C-terminus (Fig 1C). The MK5960 and MK5988 strains exhibited reversible GuHCl sensitivity (Fig 3A).

Overexpression of a dominant-negative *HSP104-KT* allele blocks the activity of Hsp104 [21, 25]. Thus, we examined the effects of overexpression of the *HSP104-KT* allele on the Mtl+ and Sor+ phenotype of the GuHCl-sensitive MK4010, MK4456, MK5960, and MK5988 strains carrying the mutated *CYC8* encoding truncated Cyc8 with polyQ at its C-terminus. Overexpression of this allele reduced the growth of these GuHCl-sensitive strains in liquid SM and SS media, but not of the GuHCl-tolerant MK4416 and MK4412 strains (Fig 3B). Moreover, deletion of *HSP104* eliminated the Mtl+ and Sor+ phenotype of the MK4010 strain, but not of the MK4416 strain; the MK4010 *hsp104Δ* ρ+ strain showed the Mtl- and Sor- phenotype, which was complemented by introduction of *HSP104* (Fig 3C). These data indicate that the Mtl+ and Sor+ phenotype of the GuHCl-sensitive MK4010, MK4456, MK5960, and MK5988 strains depends on Hsp104. The Sor+ and Mtl+ phenotype of the MK4010 and MK4456 strains were complemented by introduction of *CYC8* on a centromeric plasmid, as for other strains with the Mtl+ and Sor+ phenotype (Fig 3D).

## Discussion

Cyc8 consists of 966 amino acids and contains a central polyQ region (Fig 1B and 1C). The codons for Q are CAG and CAA; thus, amber (TAG) and ochre (TAA) nonsense mutations could occur through C to T substitutions in these codons (Fig 1C), and such a nonsense mutation (amber or ochre) could produce a truncated Cyc8 with polyQ at its C-terminus. In this study, we found that a spontaneous mutation in *TUP1* or *CYC8* conferred the ability to assimilate both mannitol and sorbitol due to dysfunction of Tup1-Cyc8. In many cases, the Mtl+ and Sor+ phenotype was tolerant to GuHCl treatment (Fig 1A and 1B and Fig 2). Moreover, we spontaneously obtained *S*. *cerevisiae* strains with the Mtl+ and Sor+ phenotype that were sensitive to GuHCl and strictly dependent on Hsp104 (Fig 3, S3 and S4 Figs).

Acquisition of the GuHCl-sensitive and Hsp104-dependent Mtl+ and Sor+ phenotype is attributable to dysfunction of the truncated Cyc8 with polyQ at its C-terminus, which is probably caused by aggregation of the truncated Cyc8 in the presence of Hsp104. This result is in accordance with reports by Kimura *et al*. [30], Patino *et al*. [31], and Krobitsch *et al*. [32] showing that Hsp104 is needed for polyQ aggregation in yeast. Kimura *et al*. found that Hsp104 is needed for conversion of polyQ tracts (N-terminal epitope-tag plus Q34 or Q80 plus C-terminal RDPAS) from a soluble form to an insoluble state [30]. Patino *et al*. reported that Sup35 was the soluble form, not aggregates, when [*PSI*^+^] cells were converted to [*psi*^-^] through deletion of *HSP104* [31]. The prion isoform of Sup35 is named [*PSI*^+^] [31]. Sup35 has a Q-rich region at its N-terminal region (S5 Fig). Krobitsch *et al*. found that aggregation of the huntingtin protein, which causes human Huntington’s disease and has a polyQ sequence in its N-terminal region, was not detected upon deletion of *HSP104* [32]. These reports indicate that Hsp104 could function to cause aggregation of some proteins with polyQ, as in the case of the truncated Cyc8 with polyQ at its C-terminus, although Hsp104 could also play a cytoprotective role in regard to aggregated heat-damaged proteins [33].

In yeast, prions are apparently propagated from generation to generation by transmitting aggregated “seeds” from mother to daughter cells via the cytoplasm, and this initiates new rounds of aggregation, as seeds are capable of immobilizing a newly synthesized protein of the same amino acid sequence and converting it into a prion state [33]. Formation of yeast prions requires a normal level of Hsp104 [33]. In this study, we observed GuHCl-sensitive and Hsp104-dependent Mtl+ and Sor+ phenotypes, which are the typical phenotypes conferred by a prion. However, these phenotypes were complemented by introduction of *CYC8* (Fig 3C). This complementation indicates that the truncated and aggregated Cyc8 with polyQ at its C-terminus is not able to convert the native form of Cyc8 into the aggregated Cyc8; i.e. the truncated and aggregated Cyc8 does not behave as a prion. The native form of Cyc8 is considered to function normally to suppress the ability to utilize mannitol and sorbitol, even in the presence of aggregated Cyc8. Thus, we concluded that the GuHCl-sensitive and Hsp104-dependent Mtl+ and Sor+ phenotype does not involve a prion.

Our results show that *S*. *cerevisiae* can acquire the ability to assimilate mannitol and sorbitol due to Tup1-Cyc8 dysfunction caused by a spontaneous mutations; i.e., due to (i) GuHCl-tolerant dysfunction of Tup1-Cyc8 caused by spontaneous mutations in *TUP1* or *CYC8* and (ii) GuHCl-sensitive and Hsp104-dependent dysfunction of Tup1-Cyc8 involving a truncated Cyc8 with polyQ at its C-terminus caused by a spontaneous nonsense mutation of *CYC8*. Mannitol is an abundant natural polyol [2] and sorbitol is the major photosynthate in *Rosaceae* species [4, 5]. Thus, it is physiologically reasonable that *S*. *cerevisiae* carries a mechanism to allow assimilation of mannitol and sorbitol for survival. However, the physiological significance of the Hsp104-dependent aspects of this assimilation remain to be established.

## Supporting information

S1 Fig Growth of BY4742, MK4412, and MK4416 strains on SM and SS solid media. Strains were streaked on the media and cultivated for 4 days.
(TIF)

S2 Fig Growth phenotype of Sor+ and Mtl+ strains. A: Sor+ strains required functional mitochondria and respiration to assimilate sorbitol. The ρ^+^ and ρ^-^ strains of parental Sor- BY4742 and the Sor+ strain (MK4416) were streaked onto the indicated media and grown for 5 days under normal (+O_2_) or anaerobic (-O_2_) conditions.
(TIF)

S3 Fig *S*. *cerevisiae* BY4742 strain spontaneously acquires the ability to assimilate sorbitol. A: Colonies on solid SS and SM media on which BY4742 WT cells (approximately 10^7^ cells per solid medium) had been spread and cultivated for 7 days. Colonies are numbered. Colony No. 2 (marked with an asterisk) showed reversible GuHCl-sensitive growth in SM and SS media and was named MK5960 strain. B: Growth of strains derived from the numbered colonies in A in solid SS and SM media. The colonies on solid SS and SM media were purified once on the same medium, pre-cultured on YPG solid medium, inoculated into 1.0 mL liquid SS and SM media to OD_600_ of 0.05, and cultivated aerobically for 2 days. White arrows indicate flocculated cells. Numbers correspond to those in A, except for No. 8 which is BY4742 WT.
(TIF)

S4 Fig GuHCl-sensitive strains. A: GuHCl-sensitive growth of the MK4010 strain. A single colony of BY4742 (WT), MK4416, or MK4010 strain grown on YPD solid medium with (GuHCl+) or without (GuHCl-) GuHCl was inoculated into 1.0 mL of SM liquid medium to OD_600_ of 0.01 and cultivated aerobically for 2 days. B: GuHCl-sensitive growth of MK5960 strain. The strain was treated and cultivated as above and also in liquid SS and SC media. C: Several colonies on solid SM-U media on which BY4742 WT strain carrying YEplac195 (approximately 10^7^ cells) had been spread and cultivated for 13 days. Of 17 colonies on SM medium, 13 were Mtl+ strains. Of the 13 strains, colonies marked with an asterisk (*) and two asterisks (**) showed reversible GuHCl-sensitive growth in SM and SS media and were named MK5986 and MK5988 strains.
(TIF)

S5 Fig Primary structure of Sup35. The sequence was obtained from the SGD (https://www.yeastgenome.org/). Glutamine (Q) residues are underlined and in bold.
(TIF)
