# Supplementary figures and images for: Hsp104-dependent ability to assimilate mannitol and sorbitol conferred by a truncated Cyc8 with a C-terminal polyglutamine in Saccharomyces cerevisiae
Source: PLoS One. 2020 Nov 11;15(11):e0242054. doi: 10.1371/journal.pone.0242054 (PMC7657529; doi:10.1371/journal.pone.0242054)

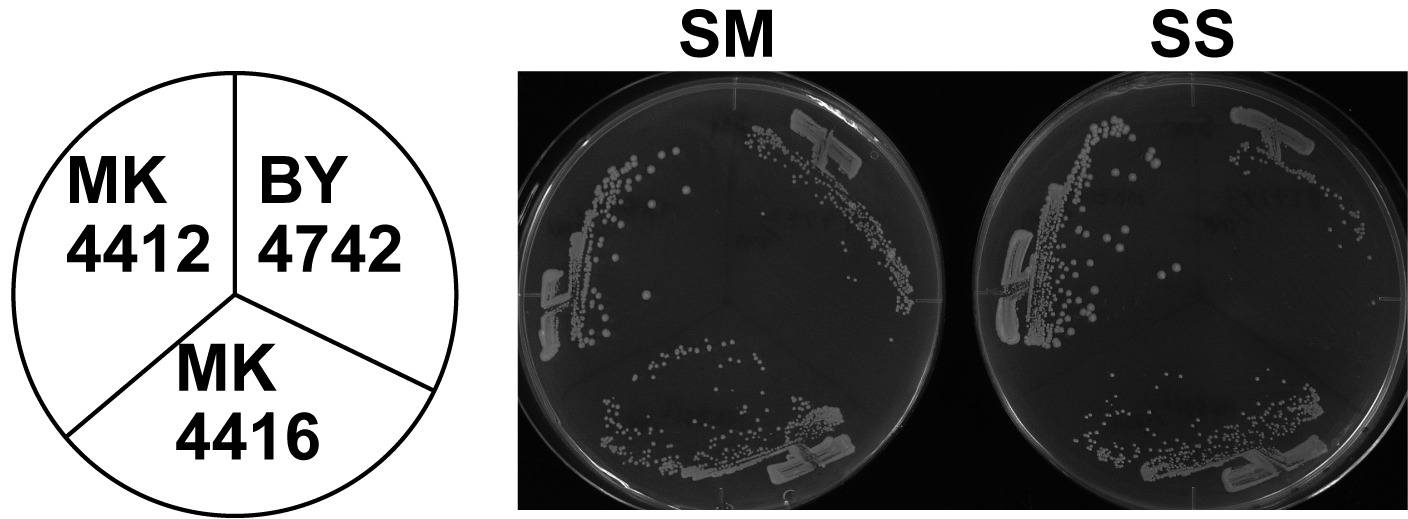

Supplement: S1 Fig — Strains were streaked on the media and cultivated for 4 days. (TIF) [file pone.0242054.s001.tif]

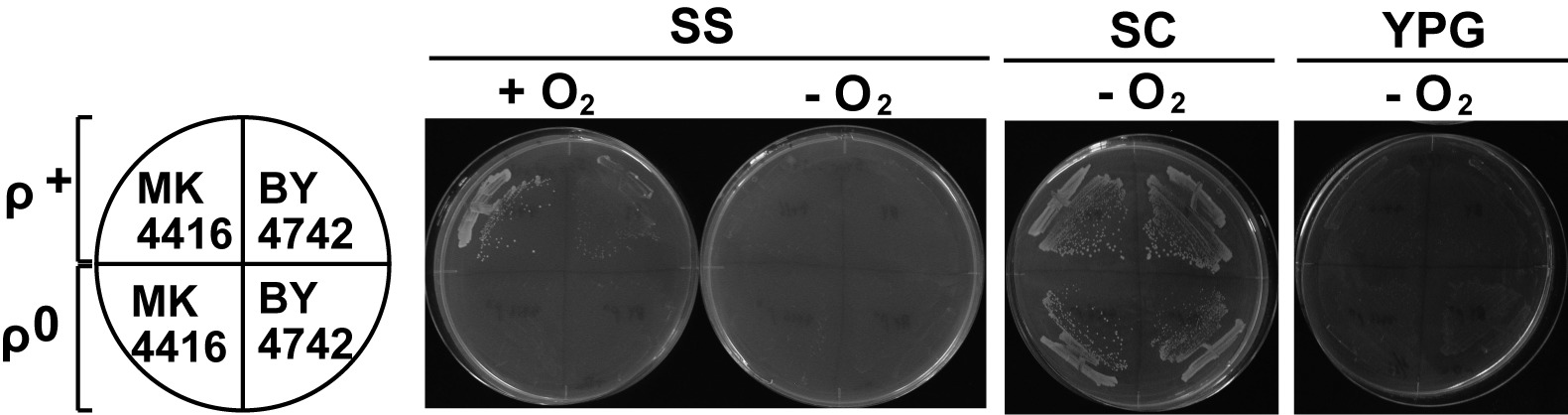

Supplement: S2 Fig — A: Sor+ strains required functional mitochondria and respiration to assimilate sorbitol. The ρ+ and ρ- strains of parental Sor- BY4742 and the Sor+ strain (MK4416) were streaked onto the indicated media and grown for 5 days under normal (+O2) or anaerobic (-O2) conditions. (TIF) [file pone.0242054.s002.tif]

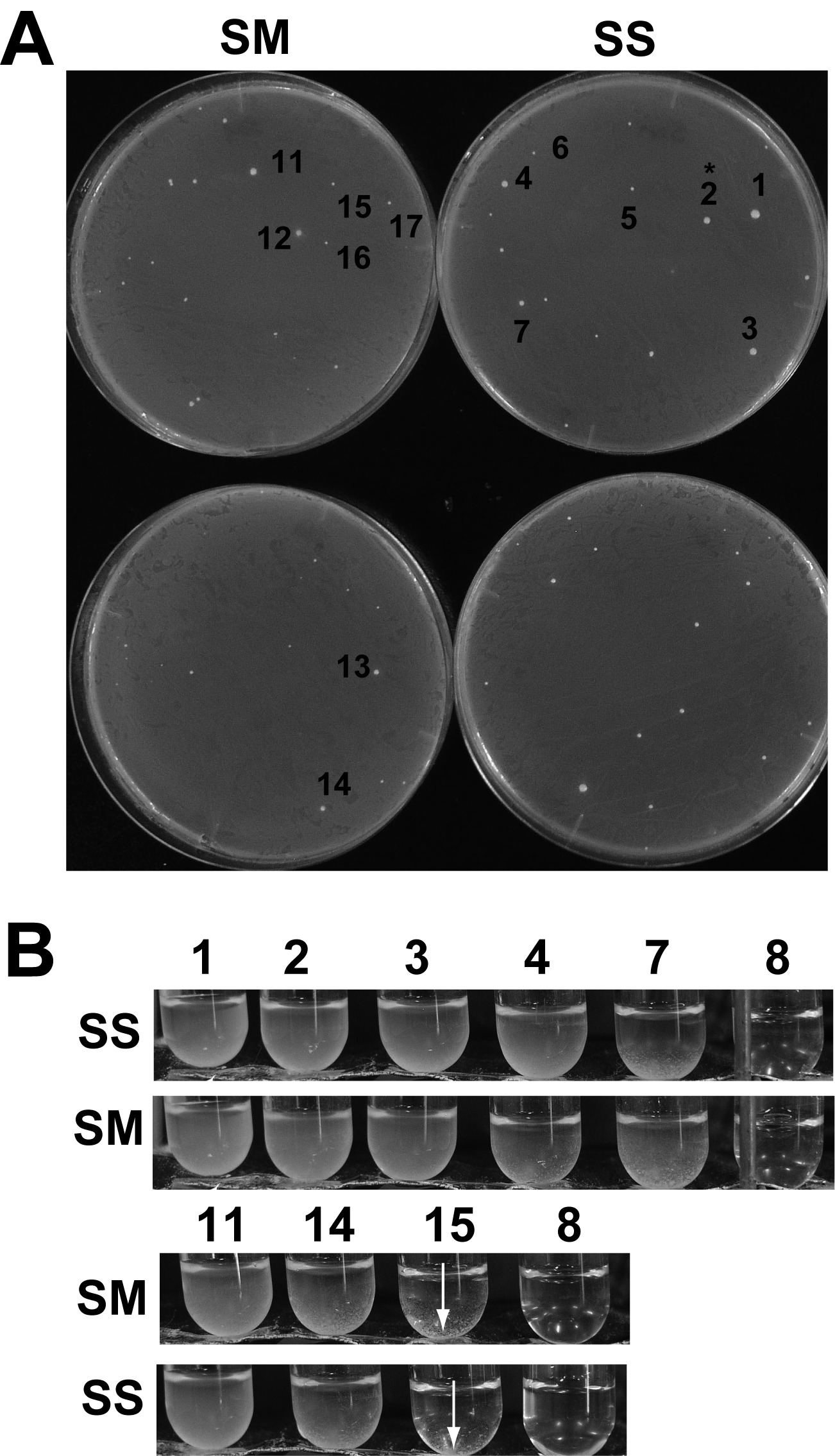

Supplement: S3 Fig — A: Colonies on solid SS and SM media on which BY4742 WT cells (approximately 107 cells per solid medium) had been spread and cultivated for 7 days. Colonies are numbered. Colony No. 2 (marked with an asterisk) showed reversible GuHCl-sensitive growth in SM and SS media and was named MK5960 strain. B: Growth of strains derived from the numbered colonies in A in solid SS and SM media. The colonies on solid SS and SM media were purified once on the same medium, pre-cultured on YPG solid medium, inoculated into 1.0 mL liquid SS and SM media to OD600 of 0.05, and cultivated aerobically for 2 days. White arrows indicate flocculated cells. Numbers correspond to those in A, except for No. 8 which is BY4742 WT. (TIF) [file pone.0242054.s003.tif]

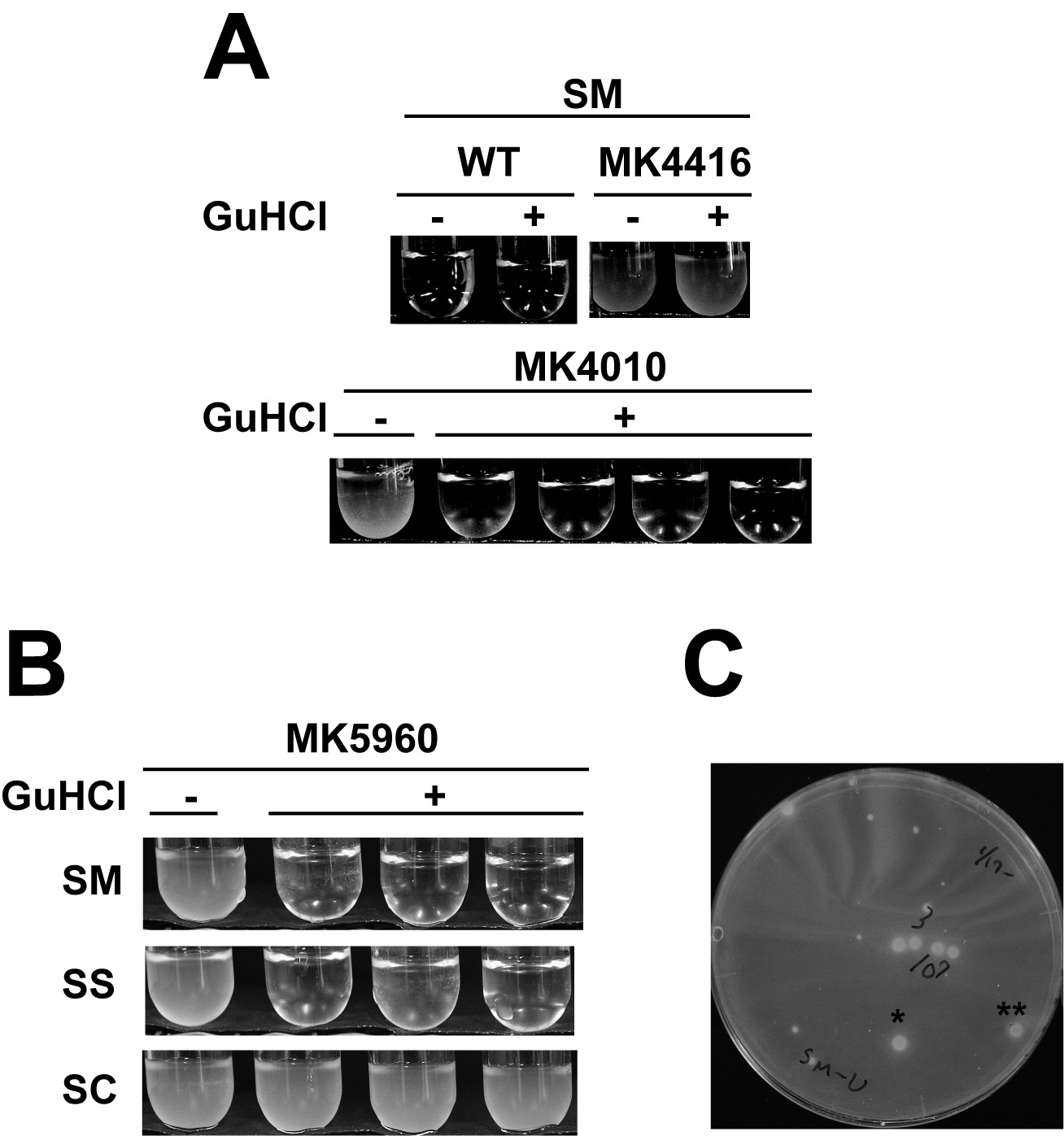

Supplement: S4 Fig — A: GuHCl-sensitive growth of the MK4010 strain. A single colony of BY4742 (WT), MK4416, or MK4010 strain grown on YPD solid medium with (GuHCl+) or without (GuHCl-) GuHCl was inoculated into 1.0 mL of SM liquid medium to OD600 of 0.01 and cultivated aerobically for 2 days. B: GuHCl-sensitive growth of MK5960 strain. The strain was treated and cultivated as above and also in liquid SS and SC media. C: Several colonies on solid SM-U media on which BY4742 WT strain carrying YEplac195 (approximately 107 cells) had been spread and cultivated for 13 days. Of 17 colonies on SM medium, 13 were Mtl+ strains. Of the 13 strains, colonies marked with an asterisk (*) and two asterisks (**) showed reversible GuHCl-sensitive growth in SM and SS media and were named MK5986 and MK5988 strains. (TIF) [file pone.0242054.s004.tif]

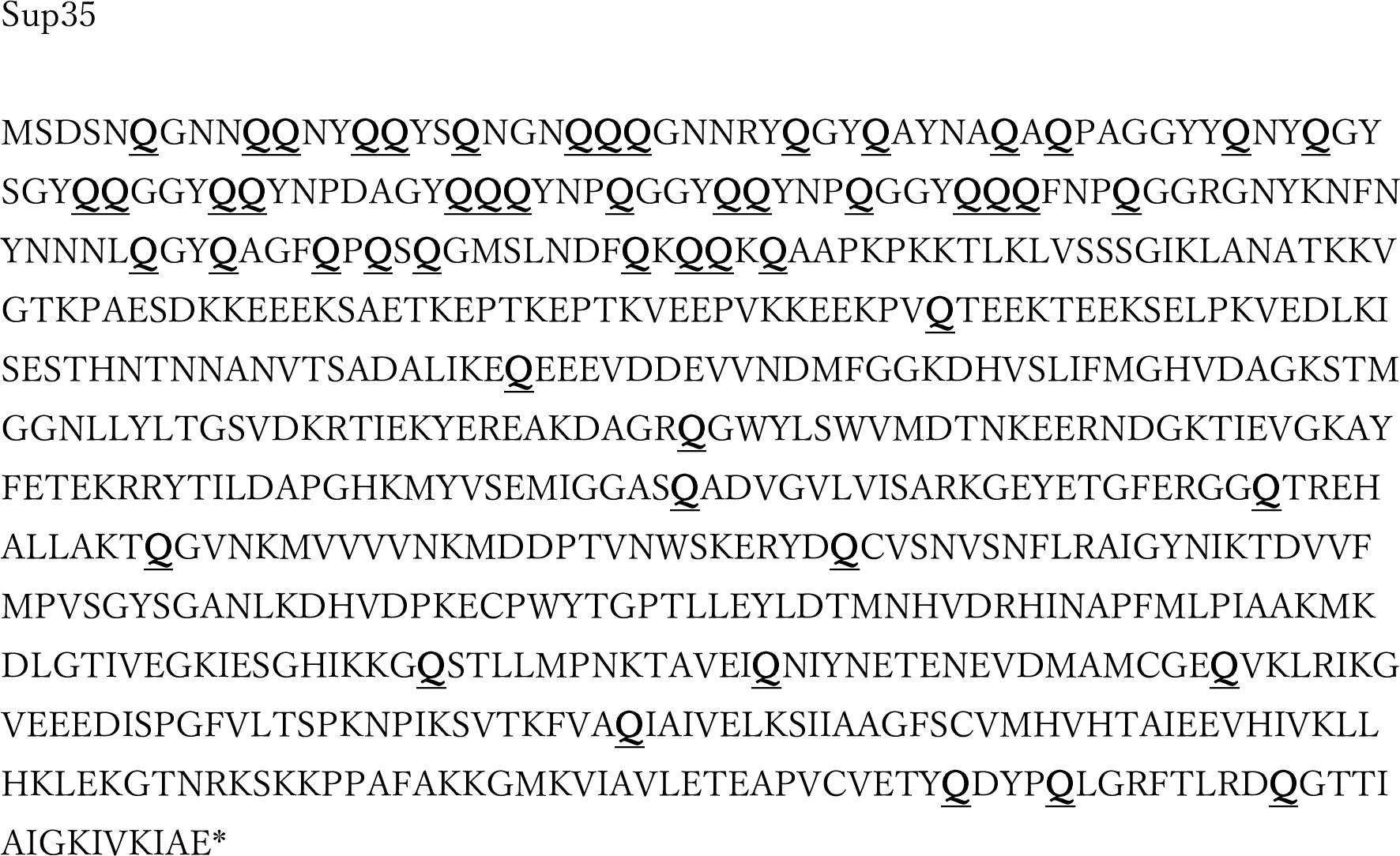

Supplement: S5 Fig — The sequence was obtained from the SGD (https://www.yeastgenome.org/). Glutamine (Q) residues are underlined and in bold. (TIF) [file pone.0242054.s005.tif]
